# Engineering glucose metabolism of *Escherichia coli* under nitrogen starvation

**DOI:** 10.1038/npjsba.2016.35

**Published:** 2017-01-05

**Authors:** Victor Chubukov, John James Desmarais, George Wang, Leanne Jade G Chan, Edward EK Baidoo, Christopher J Petzold, Jay D Keasling, Aindrila Mukhopadhyay

**Affiliations:** 1 Joint BioEnergy Institute, Emeryville, CA, USA; 2 Biological Systems and Engineering Division, Lawrence Berkeley National Laboratory, Berkeley, CA, USA; 3 Department of Chemical & Biomolecular Engineering, University of California, Berkeley, CA, USA; 4 Department of Bioengineering, University of California, Berkeley, CA, USA; 5 Novo Nordisk Foundation Center for Biosustainability, Technical University of Denmark, Hørsholm, Denmark

## Abstract

A major aspect of microbial metabolic engineering is the development of chassis hosts that have favorable global metabolic phenotypes, and can be further engineered to produce a variety of compounds. In this work, we focus on the problem of decoupling growth and production in the model bacterium *Escherichia coli*, and in particular on the maintenance of active metabolism during nitrogen-limited stationary phase. We find that by overexpressing the enzyme PtsI, a component of the glucose uptake system that is inhibited by α-ketoglutarate during nitrogen limitation, we are able to achieve a fourfold increase in metabolic rates. Alternative systems were also tested: chimeric PtsI proteins hypothesized to be insensitive to α-ketoglutarate did not improve metabolic rates under the conditions tested, whereas systems based on the galactose permease GalP suffered from energy stress and extreme sensitivity to expression level. Overexpression of PtsI is likely to be a useful arrow in the metabolic engineer’s quiver as productivity of engineered pathways becomes limited by central metabolic rates during stationary phase production processes.

## Introduction

The promise of metabolic engineering lies in the production of a wide variety of chemicals from renewable feedstocks using microbial catalysts. Production of liquid fuels or commodity chemicals such as plastic monomers via this carbon-neutral process could significantly decrease the deleterious effects of fossil fuel consumption and associated climate effects. Driven by advances in synthetic biology techniques, the last decade has seen an enormous increase in the variety of molecules that can be produced by cultivatable microbes. Nevertheless, the number of molecules that can be produced at industrially relevant cost and scale remains significantly lower, especially when considering bulk chemicals.^1^ Although many factors contribute to this gap, one key point is that the ability to manipulate individual pathways has greatly outpaced the ability to manipulate the global regulatory machinery that controls the cellular metabolic and physiological state.^2^ Such global engineering is crucial to ensure the most efficient allocation of carbon and energy resources to the desired product. Equally critically, global engineering of cellular physiology is required to ensure that cells maintain predictable behavior in the complex and heterogeneous environment of the large-scale bioreactor.

In particular, an industrial bioproduction process will inevitably create selection pressure for cells that are able to grow without diverting carbon and energy to the engineered pathway. Non-producing subpopulations immediately detract from productivity and yield, and a non-producing mutant that takes over the population leads to collapse of the entire process. There are generally two strategies to overcome this challenge. One is growth-coupling, where the metabolic pathway to produce the desired product also produces a byproduct crucial for growth^3,4^—a common case is regeneration of oxidized cofactors by reductive pathways.^5,6^ Unfortunately, not all pathways can be coupled to growth, and even in successful cases, a large amount of carbon goes to biomass instead of product. The alternative is growth decoupling, in which the growth and production phases are separated. This has the advantage that during the production phase, 100% of carbon can theoretically be directed to product synthesis. For production of hydrocarbons such as biofuels, one simple method to achieve a separate production phase is to limit a non-carbon nutrient that is essential for growth. Popular choices include nitrogen^7^ and phosphate,^8^ though sulfate^9^ or essential metals like Mg, Zn or Fe are also occasionally used. Complex synthetic circuits to arrest growth in response to a specific signal are also gaining in popularity.^10^

The downside of the decoupling approach is that the lack of growth also removes selective pressure for active metabolism, and fast growing microbes such as *E. coli,* which are ubiquitous chassis hosts for metabolic engineering, tend to slow and eventually shut down their metabolism during the non-growth phase.^11^ Thus, finding ways to alter the global regulation of cellular metabolism to maintain metabolically active cells during stationary phase is a major goal of present-day metabolic engineering. In this study, we focus on engineering metabolic activity during nitrogen starvation in the model bacterium *E. coli*. Nitrogen starvation is a particularly attractive condition for industrial bioprocesses—nitrogen comprises a large fraction of biomass, its extracellular levels are easy to control, and unlike phosphate, it is not a critical component of respiration and energy transduction. However, *E. coli*, along with many other microbes, has evolved careful regulation that tightly links its central carbon and nitrogen metabolism.^12–14^ A previous study showed that glucose uptake rates in *E. coli* under nitrogen starvation decreased to about 5% of the rates observed under exponential growth, a more drastic decrease than any other condition tested.^11^

Nitrogen starvation typically leads to drastic decreases in cellular amino acids, particularly glutamine, and a concurrent increase in alpha-ketoglutarate (αKG). Glutamine and αKG serve as two of the most important signaling molecules to coordinate carbon and nitrogen metabolism, acting both as direct modulators of metabolic enzymes as well as modulators of transcription factors and other regulators.^15^ A particularly critical interaction linking nitrogen availability to glucose uptake is the inhibition of the PtsI protein by αKG.^16^ PtsI, also known as Enzyme I of the phosphotransferase glucose uptake system, is responsible for converting phosphoenolpyruvate (PEP) to pyruvate, with the phosphate group transferred to an imported glucose molecule via a cascade of several enzymes. Structural and biochemical studies have characterized this interaction in detail, showing that αKG competes for the PEP-binding site on PtsI, and determining *K*_m_ and *K*_d_ values that are consistent with *in vivo* concentrations of PEP and αKG.^17,18^

To further understand regulation of glucose metabolism under nitrogen starvation and to engineer strains with enhanced metabolic activity in this condition, we engineered *E. coli* to use several alternative glucose uptake systems. This included overexpression of wild-type PtsI, expression of a chimeric version of the PtsI protein and overexpression of the galactose permease GalP and glucokinase Glk. Surprisingly, we found that simple overexpression of wild-type PtsI was effective in significantly increasing glucose uptake during nitrogen starvation. Cells overexpressing PtsI metabolized glucose at approximately fourfold higher rates than wild-type cells, and maintained metabolite concentrations typical of metabolically active cells, such as high fructose-bis-phosphate levels and high adenylate energy charge. To demonstrate the potential of the growth decoupling approach and to apply our findings to a production strain, we investigated fatty alcohol production during nitrogen starvation in engineered *E. coli* strains. Additional expression of PtsI did not alter the overall production parameters, presumably due to other rate-limiting steps in the pathway. More significantly, we found increased product yield in nitrogen starvation conditions compared with exponential growth, and found that the higher glucose uptake rate of PtsI-overexpressing cells was maintained even in stationary phase.

## Results

**Figure 1:**
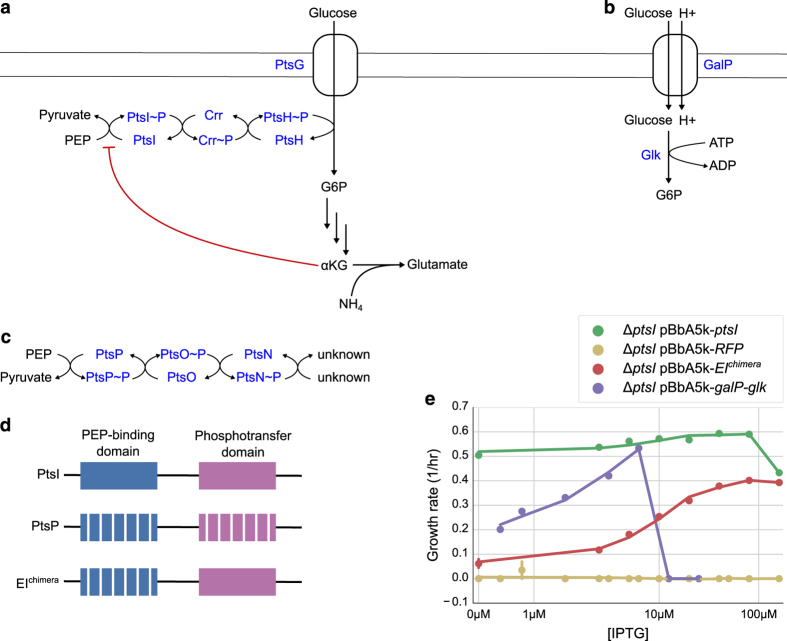
Alternative glucose uptake systems in *E. coli.* (**a**) Schematic of the PTS system used for concurrent glucose uptake and phosphorylation under most conditions. The first enzyme of the phosphotransfer cascade, PtsI, is inhibited by αKG, which accumulates under nitrogen-limited conditions. (**b**) The galactose permease GalP typically transports galactose, but can also transport glucose if overexpressed. The glucokinase enzyme Glk then phosphorylates intracellular glucose to glucose-6-phosphate. (**c**) The paralogous PtsNOP system, known as the ‘Nitrogen PTS’ can also use PEP as a phosphate donor, although the final phosphate acceptor is unknown. (**d**) A chimeric protein composed of PtsP PEP-binding domain and the PtsI phosphotransfer domain was proposed to mediate PTS glucose uptake without αKG inhibition. (**e**) Growth rates of *ΔptsI* strains expressing various alternative glucose uptake systems or RFP control.

Our primary attempt to overcome the inhibition of PtsI by αKG centered on a chimeric protein on the basis of the paralogous PTS system in *E. coli* consisting of PtsNOP. Often referred to as the ‘nitrogen PTS’, it was recently shown that the association between the PtsNOP system and nitrogen availability in early experiments was in fact an artifact related to a mutation in *ilvG.*^19^ The exact function of PtsNOP remains unclear, but appears to be related to potassium uptake. Importantly, however, PtsP, the paralog of PtsI, can bind and use PEP as a phosphate donor, and this activity appears to not be inhibited by αKG.^20^ Thus, we constructed a hybrid enzyme, consisting of the N-terminal domain (residues 1–249) from PtsP and the C-terminal domain (residues 412–748) from PtsI (Figures 1c and d). This strategy was also proposed in ref. 17.

We expressed the hybrid EI^chimera^ in an *E. coli* Δ*ptsI* background using an IPTG-inducible promoter on a medium-copy plasmid. As controls, we expressed (a) red fluorescent protein (RFP), (b) the wild-type PtsI enzyme or (c) an alternative system, consisting of the galactose permease GalP and glucokinase Glk, which has also been shown to be able to mediate glucose uptake in the absence of a functioning PTS system^21^ (Figures 1a and b). Encouragingly, the chimeric enzyme was able to restore growth on glucose minimal medium, with the rescue dependent on increasing amounts of inducer and reaching a maximum growth rate of *μ*=0.4/h, or about 66% of wild-type growth rates. As expected, strains expressing RFP did not grow at all, whereas strains expressing wild-type PtsI grew at approximately wild-type rates. Strains expressing GalP-Glk reached almost wild-type growth rates, but were extremely sensitive to the concentration of inducer, with optimal growth achieved in a very narrow window of IPTG concentration (Figure 1e).

**Figure 2:**
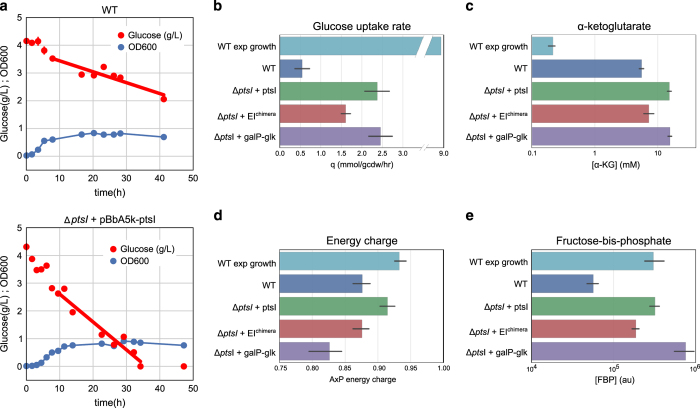
Phenotypes of *E. coli* expressing alternative glucose uptake systems under nitrogen starvation (except top bar of plots **b**–**e**). (**a**) Optical density and glucose uptake for wild-type and PtsI-overexpressing *E. coli.* (**b**–**e**) Phenotypic metrics of *E. coli* expressing various glucose uptake systems: glucose uptake rate (**b**), intracellular α-ketoglutarate (**c**), intracellular adenosine energy charge, defined as ([ATP]+0.5[ADP])/([ATP]+[ADP]+[AMP]) (**d**) and intracellular fructose-bis-phosphate, whose concentration is typically correlated with glycolytic flux (**e**). Error bars are 95% confidence intervals on the basis of two biological replicates x three technical replicates.

We next set out to investigate whether any of the strains showed increased metabolic activity, quantified by glucose uptake rate, during nitrogen starvation. Expression of the chimeric protein resulted in about a 2.5-fold increase compared with wild-type cells. Surprisingly, however, strains expressing wild-type PtsI saw an even larger increase in glucose uptake rates, about a fourfold increase over wild-type rates. A similar rate was observed in the GalP-Glk expressing strains, which do not rely on any enzymes known to be regulated by αKG (Figures 2a and b; Supplementary Table 1).

The fact that the chimeric protein fulfilled the role of PtsI for glucose uptake, but did not lead to an increase in glucose uptake during nitrogen starvation (compared with expression of wild-type PtsI) was surprising. Nevertheless, the finding that expression of wild-type PtsI was sufficient to significantly increase glucose uptake during nitrogen starvation was promising, and we focused on this phenotype. We examined the behavior across a wide range of inducer concentrations and found that only mild overexpression of PtsI was required (Supplementary Figure 1). No obvious undesired phenotypes (such as reduced growth rates) were observed from this level of PtsI overexpression. We also quantified cell lysis by measuring released DNA, and verified that the glucose uptake was not due to increased rates of lysis and cannibalistic growth, but rather to truly metabolically active but non-growing cells (Supplementary Figure 2).

We further investigated the metabolically active phenotype of PtsI-overexpressing cells by examining key extracellular and intracellular metabolites. First, we examined extracellular product secretion and found remarkably little accumulation of typical fermentation products, such as acetate, lactate or pyruvate (Supplementary Figure 3), suggesting that the vast majority of carbon is combusted to CO_2_ via an active TCA cycle. A significant contribution to carbon storage metabolites is unlikely—the stationary phase cells consumed several times their own weight in glucose, whereas reported estimates of glycogen content in *E. coli* rarely exceed 20%.^22^ Second, analysis of the intracellular metabolites showed that αKG concentrations in PtsI-overexpressing cells were even higher than in wild type (Figure 2c). These data show that the increased metabolic rates are not caused by preventing the accumulation of αKG but rather by overcoming it through higher expression of PtsI. We also observed increased fructose-bis-phosphate concentrations in engineered cells (Figure 2e), which were consistent with increased glycolytic activity, as fructose-bis-phosphate concentration has been shown to be directly coupled to the glycolytic flux rate under many conditions.^23^

Finally, we found that strains overexpressing PtsI had an adenosine energy charge as high as that found in cells growing exponentially in non-limiting medium (Figure 2d). Adenosine energy charge is a common measure of the energy state of the cell,^24^ and further points to a highly metabolically active yet non-growing cell. Although the full combustion of glucose to CO_2_ is not directly advantageous for metabolic engineering, the fact that the cell is able to run a full metabolic program to maintain a steady supply of energy is crucial for the success of further metabolic modification.

In contrast, strains overexpressing GalP-Glk, while maintaining high glucose uptake rates, showed significantly decreased energy charge. Impaired respiration and energy-related stresses are common effects from overexpression of membrane proteins^25,26^ and the sensitivity of strains expressing GalP-Glk to the precise inducer concentration is also consistent with a delicate balance between GalP function and membrane stress. An additional factor impacting the energy state may be the fact that import of glucose through the GalP system requires import of a proton along the electrochemical gradient, which translates to about one-third of an ATP in terms of energy cost.

**Figure 3:**
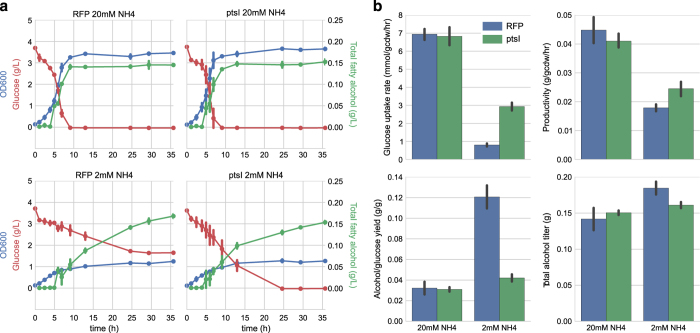
Fatty alcohol production in nitrogen-starved *E. coli*. (**a**) Time course of optical density, glucose and total fatty alcohols in wild type (left) or PtsI-overexpressing (right) cells and either carbon-limited (top) or nitrogen-limited (bottom) media. Error bars are 95% confidence intervals based on two biological replicates. (**b**) Production metrics (glucose uptake rate and fatty alcohol yield, titer and productivity) for the strains and conditions above. Error bars are 95% confidence intervals based on four biological replicates (two cultures on two different days).

Given the promising phenotype of PtsI-overexpressing *E. coli*, we aimed to apply this strain to production of valuable compounds. We focused on production of long-chain fatty alcohols, which were successfully made at g/l titers in ref. 27. We transformed the Δ*fadE* strain (deficient in fatty acid degradation), with two plasmids—one expressing PtsI on a low-copy plasmid, and one expressing the acyl-coA/acyl-ACP reductase Maqu2220 from *Marinobacter aquaeolei* on a high-copy plasmid. We grew this strain, along with a control expressing RFP instead of PtsI, in either carbon-limited medium (4 g/l glucose and 20 m mol/l ammonium) or nitrogen-limited medium (4 g/l glucose and 2 m mol/l ammonium). In carbon-limited medium, expression of PtsI had no effect on growth, glucose uptake or fatty alcohol production. Both strains produced ~150 mg/l fatty alcohol during batch growth, for a yield of ~0.03 g/(g glucose) (Figures 3a and b; Supplementary Table 2).

Next we tested if production of fatty alcohols would be maintained during nitrogen starvation, as this cannot be predicted from exponential phase production profiles. Although nitrogen is not directly required for the product, many of the steps of fatty acid synthesis are subject to complex regulation, and synthesis may be inhibited in the absence of growth. Encouragingly, we found that production of fatty alcohols was maintained throughout the starvation phase. In the RFP-expressing strain, the lack of carbon diversion to biomass resulted in a yield of 0.12 g/g, about fourfold higher than the yield observed under the carbon-limited condition and close to 50% of the theoretical maximum yield (Figure 3b).

Given the continued production of fatty alcohols throughout the nitrogen starvation phase, we tested if the increased metabolic rate displayed by the PtsI-overexpressing cells would translate into increased productivity. However, despite the fact that the fourfold higher rate of glucose consumption remained, it did not translate into increased productivity (Figure 3b). Instead, the productivity was virtually identical to the RFP-overexpressing strain, suggesting that the major limitation in fatty alcohol flux was in the pathway from acetyl-coA to the final product, as discussed in more detail in the next section.

## Discussion

As described in the introduction, increasing the metabolic rate of model organisms such as *E. coli* under non-growth conditions could lead to significant improvements in the productivity of bioprocesses. A large number of very diverse strategies have been pursued in developing a non-growing but metabolically active cell—examples include synthetically arresting DNA replication^28^ or transcription,^10^ repurposing toxin-antitoxin and pheromone-sensing systems,^29,30^ and evolution in oscillating environments.^31^ In this study, we pursued a rational engineering approach to increasing the metabolic rate under nitrogen starvation, which is a robust and easily implementable industrial process condition.

On the basis of competitive inhibition of PtsI by αKG being the key allosteric interaction attenuating glucose uptake under nitrogen starvation, we investigated a number of modifications of the glucose uptake system. We first sought to prevent the inhibition of glucose uptake by αKG by expressing a chimeric variant of Enzyme I, consisting of the C-terminal domain of PtsI and the N-terminal domain of the paralog PtsP. This was on the basis of previous reports that PtsP activity is not inhibited by αKG. The chimeric enzyme was found to be functional and allowed growth on glucose at ~66% of normal growth rates. However, we observed that the glucose uptake rate under nitrogen starvation was only mildly increased compared with wild type, and not as high as the rate obtained by overexpressing wild-type PtsI. Although it is difficult to speculate on the exact properties of the chimeric protein, the most likely explanation is that the PtsP enzyme is in fact also sensitive to αKG. We note that *in vitro* experiments in the original publication^20^ were performed at 10 mmol/l PEP, whereas typical intracellular concentrations of PEP in *E. coli* are in the 100 μmol/l range.^11,32^ Since the αKG inhibition is competitive with PEP, it is unlikely that any effect would have been observed at such a high PEP concentration.

Despite the fact that the chimeric protein did not result in the desired phenotype, we found that the simple solution of overexpressing wild-type PtsI was sufficient to increase the metabolic rate of *E. coli* by approximately fourfold. This occurred despite the fact that αKG concentrations in this strain were even higher than in wild-type cells. From a metabolic control analysis viewpoint, this shows that PtsI has a high flux control coefficient for glycolysis. However, as typically occurs, as PtsI expression was increased past a certain point, there was no further increase in flux, meaning that control had shifted to other enzymes of the pathway. Understanding the other regulatory interactions that control glycolytic flux in nitrogen starvation would be crucial to further engineering.

We found that only mild overexpression of the wild-type PtsI enzyme was sufficient to obtain the desired phenotype. Forty micromolar IPTG with the lacUV5 promoter and a medium-copy plasmid was more than sufficient, and mass spectrometry quantification suggested that this resulted in about a threefold increase in PtsI expression compared with the wild type (Supplementary Figure 4). However, the phenotype was robust across a wide range of inducer concentrations (Supplementary Figure 1). Meanwhile, we found that the alternative glucose uptake system encoded by *galP* and *glk* was extremely sensitive to inducer concentration, with a sharp threshold between optimal growth and no growth. This is likely due to the deleterious effects associated with expressing the transmembrane protein GalP at high levels (which is necessary to compensate for its relatively low k_cat_).^33^ Furthermore, glucose uptake by GalP is slightly less energetically efficient due to the co-transport of a proton along the electrochemical gradient. The combination of all these factors make *galP-glk* a more challenging system for global engineering of glucose import, despite the fact that it has been used successfully in a few cases.^21,33^

To demonstrate the value of production under nitrogen starvation conditions, and to further investigate the effects of PtsI overexpression in this context, we explored the productivity of a C_12_–C_16_ fatty alcohol production system.^27^ In wild-type cells combined with the fatty alcohol production system, we were successful in obtaining continued production of fatty alcohols for at least 24 h during nitrogen starvation, and as predicted, yield (g product/g substrate) increased significantly in this phase compared with carbon-limited batch growth. This effect shows the promise of separate growth and production phases in microbial manufacturing processes. Since glucose consumption rates remained low, we hypothesized that the PtsI-overexpressing strain would maintain higher yield while also increasing productivity via increased glucose uptake rates. However, the productivity in the PtsI-overexpressing strain was virtually identical to control strains expressing RFP. This similarity suggests that in this strain, the flux-limiting steps for fatty alcohol production during nitrogen starvation were not in glycolysis but rather in the fatty acid synthesis pathway or the heterologous acyl-coA/acyl-ACP reductase. As a result, even the very low glucose uptake rate observed in wild-type *E. coli* under nitrogen starvation is sufficient for maximum flux through the pathway.

It is certain, however, that as engineered metabolic pathways become more efficient, they will run into the metabolic limits of non-growing cells and the traditional route of engineering a carbon sink by introducing a heterologous pathway may not be sufficient to overcome these limits. In this study, the glucose consumption rates during nitrogen starvation were essentially identical between wild-type and fatty alcohol production strains. On the other hand, increasing carbon availability by overexpressing PtsI requires an efficient downstream pathway to translate into an increase in productivity. A combination of these ‘push’ and ‘pull’ approaches will be necessary for progress in microbial metabolic engineering. In addition, our work underscores the importance of concurrent process engineering and strain development. Different bioproduction processes will impose different environmental demands, and understanding their effects on metabolism is crucial for efficient strain design. In the context of a nitrogen-limited process, which is a promising approach for hydrocarbon production, we have shown that overexpression of PtsI will be a useful arrow in the metabolic engineer’s quiver.

## Materials and methods

### Strains and media

*E. coli* BW25113 (referred to as wild type) and *ΔptsI* strains was obtained from the KEIO collection and the kanamycin resistance marker was removed by FLT-FRT recombination.^34^ PtsI, EI^chimera^ and GalP-Glk were cloned behind the IPTG-inducible lacUV5 promoter in the pBbA5k vector.^35^ J5 DNA assembly design software^36^ was used for all primer and construct design, and plasmids were assembled using the Gibson method.^37^ Sequences of all plasmids are available at http://public-registry.jbei.org,^38^ which also provides a mechanism for researchers to request strains.

M9 medium with 4 g/l glucose was used for all experiments; the composition was identical to ref. 11 except that 10 μ mol/l FeSO_4_ was used in place of FeCl_2_. NH_4_Cl was added to 20 mmol/l in for the non-nitrogen-limited conditions, and to 2 mmol/l for the nitrogen-limited conditions. For experiments to characterize glucose uptake systems, cells were maintained in steady state exponential growth with constant concentration of inducer for at least 10 generations before nitrogen was allowed to be depleted. Unless specified otherwise, 40 μ mol/l IPTG was used for RFP, PtsI or EI^chimera^ strains, and 5 μ mol/l IPTG was used for GalP-Glk strains.

For fatty alcohol production, the PtsI expression construct was transferred to a low-copy chloramphenicol resistance plasmid (pBbS5c). This was transformed into MG1655 *ΔfadE*^39^ along with the pBbB5k-Maqu2220 plasmid, which contained the *E. coli* codon-optimized Maqu2220 acyl-CoA/acyl-ACP reductase from *M. aquaeolei* under the *lacUV5* promoter.^27^ For production experiments, cells were maintained in steady state exponential growth for at least 10 generations, then IPTG was added to 500 μ mol/l at OD600≈0.1.

### Analytical methods

Extracellular glucose was measured using an enzymatic assay (GOPOD kit, Megazyme, Bray, Ireland). Extracellular acetate, lactate and pyruvate were measured by high pressure liquid chromatography with a UV absorbance detector (Agilent, Santa Clara, CA, USA). For growing cells, yields of consumed glucose/gcdw were calculated by linear regression, and the specific uptake rate was calculated by multiplying by the calculated growth rate. For all calculations, a constant of 0.41 gcdw/(L×OD_600_) was assumed.

For intracellular metabolite extracts, samples were collected by vacuum filtering 1 ml of culture onto a 0.22 μm Durapore filter (Millipore, Billerica, MA, USA) and immediately placing into 4 ml of cold (−20 °C) 40/40/20 (v/v/v) acetonitrile/methanol/water mixture. Sodium glutarate was added as an internal standard. Cell debris was pelleted by centrifugation at −9 °C and the supernatants were frozen in liquid nitrogen and then lyophilized to dryness. Samples were resuspended in 50 μl of cold 50/50 methanol/water for mass spectrometry analysis. UHPLC-TOF-MS was performed on an Agilent 6550 Q-TOF in negative mode. Absolute quantification of ATP, ADP, AMP and α-ketoglutarate was obtained by comparison to a calibration curve of pure standards (Sigma-Aldrich, St Louis, MO, USA).

For analysis of fatty alcohols, 500 μl of cell culture was extracted with 500 μl of 2:1 (v/v) chloroform/methanol containing 100 mg/l pentadecanol as an internal standard (odd chain lengths are not produced by *E. coli*). Samples were vortexed for 5 min and centrifuged at room temperature; 100 μl of the chloroform phase was collected and dried overnight. Samples were resuspended in 100 μl hexanes and analyzed by GC-MS. Total abundance was calculated on the basis of the ratio between the sum of the C_14_, C_16_ and C_18_ peaks (including saturated and monounsaturated species) to the C_15_ peak.

Proteomic samples were prepared as described previously^40^ and analyzed as described in ref. 41.
