# Supplementary material for: Engineering glucose metabolism of Escherichia coli under nitrogen starvation
Source: NPJ Syst Biol Appl. 2017 Jan 5;3:16035–. doi: 10.1038/npjsba.2016.35 (PMC5516864; doi:10.1038/npjsba.2016.35)
Supplement: Supplementary Figures [file npjsba201635-s1.doc]

**Supplementary Figures**

**
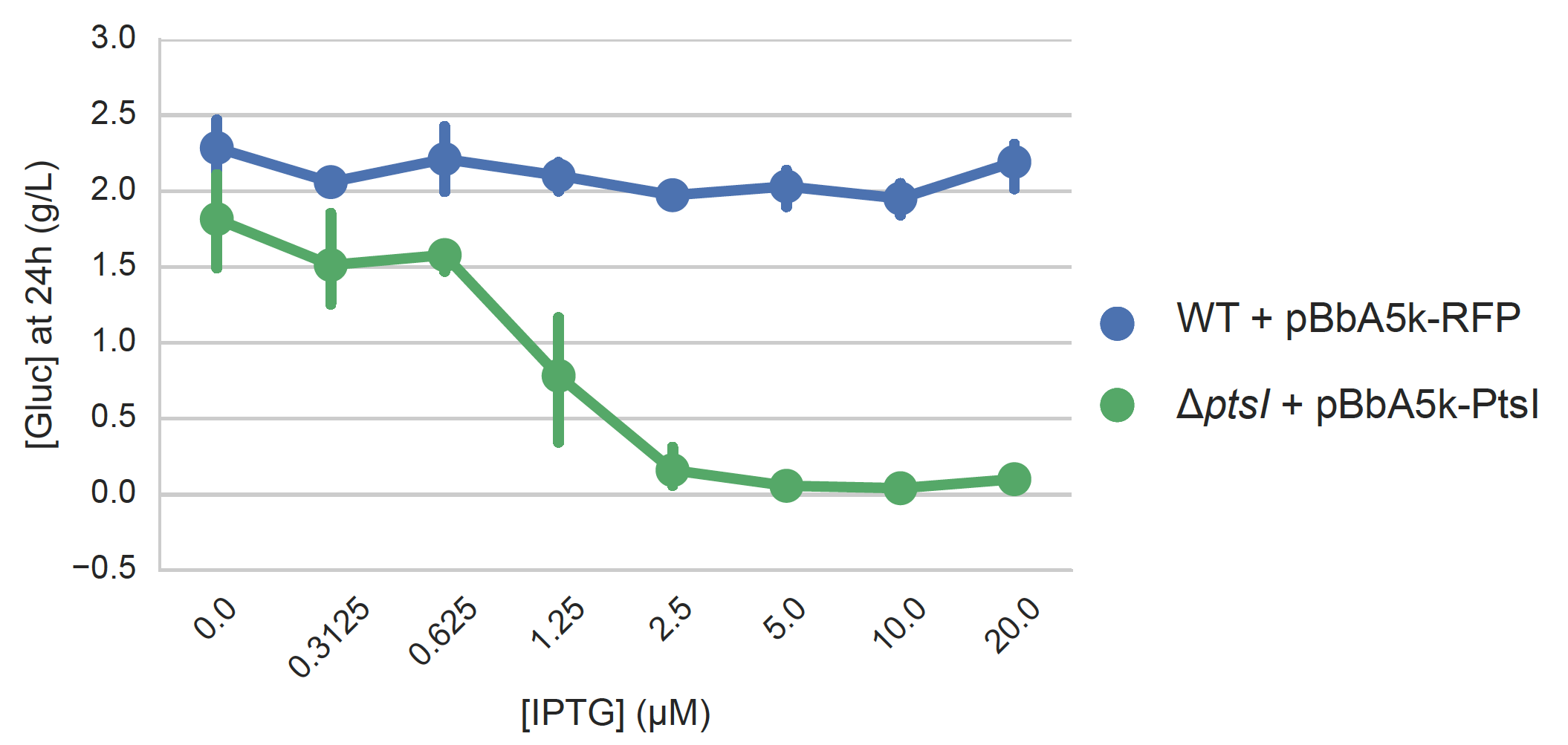
**

Supplementary Figure 1. Glucose consumption of PtsI-overexpressing *E. coli* as a function of inducer concentration. Strains were inoculated at identical densities and residual glucose was measured at 24 hours. Error bars are 95% confidence intervals based on 4 biological replicates.


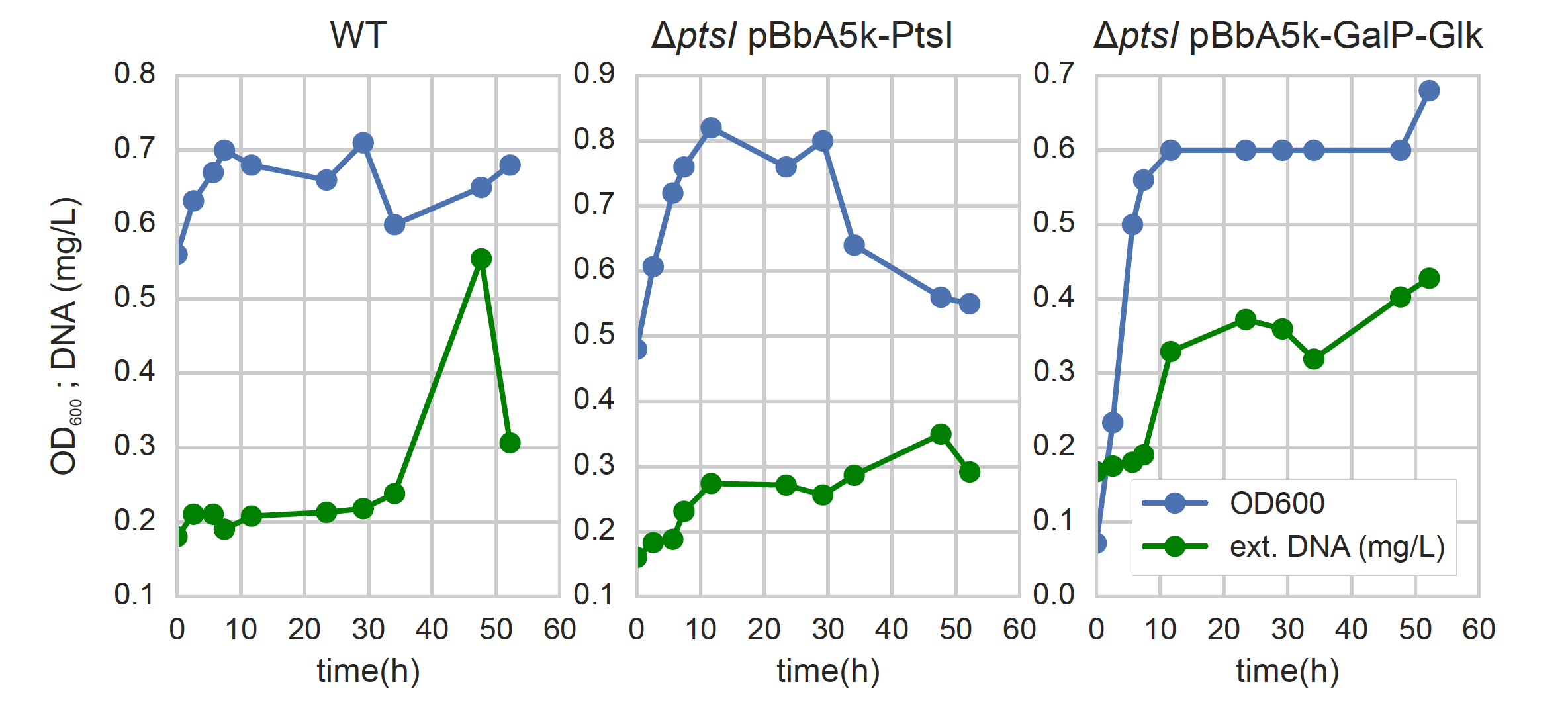


Supplementary Figure 2. Estimation of cell lysis by measurement of extracellular DNA. Spent medium samples were incubated with GelRed nucleic acid stain, followed by fluorescence measurement (ex: 280nm, em: 600nm). A calibration curve was calculated using pure standard DNA and additional positive controls were generated by boiling known quantities of *E. coli* cells (not shown). Assuming a DNA content of approximately 2% of cell dry weight, 0.25mg/L DNA would suggest that at most 5% of cells have lysed over the time course, which would have negligible impact on carbon and nitrogen availability. A contribution from RNA would imply that the lysed fraction is even lower.


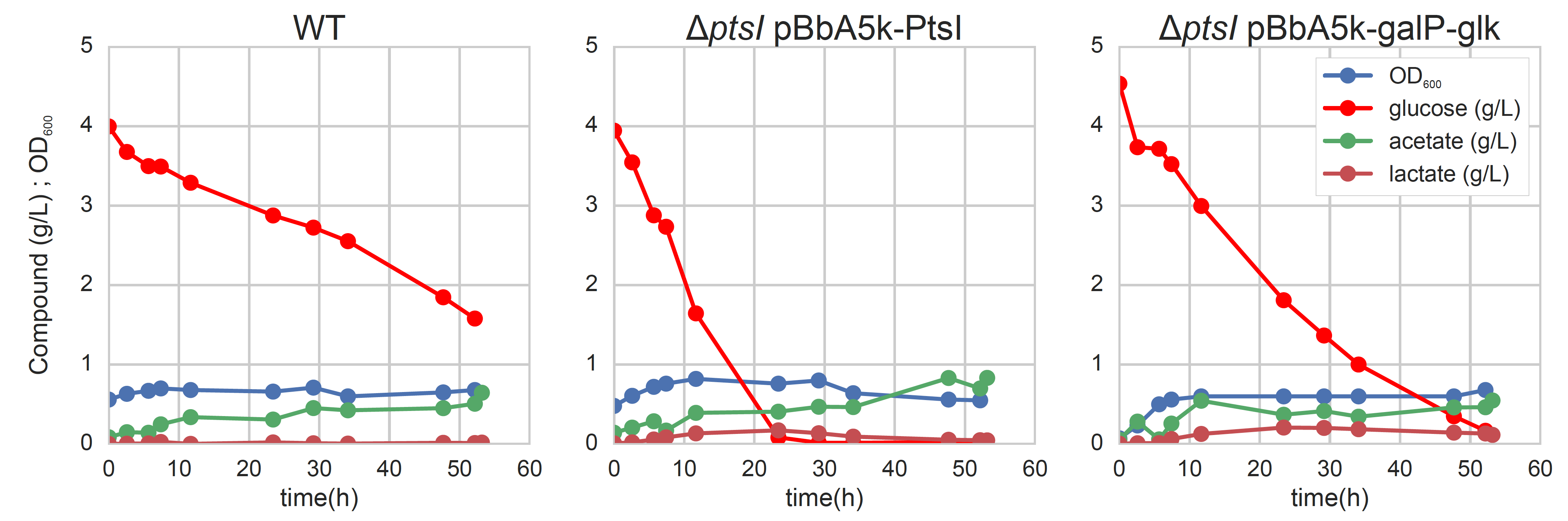


Supplementary Figure 3. Quantification of secreted carbon products in PtsI or GalP-Glk overexpressing *E. coli.* Overall, fermentation products such as lactate and acetate accounted for a small fraction of consumed carbon. Pyruvate, formate, succinate, and α-ketoglutarate were also measured but no medium accumulation was observed.


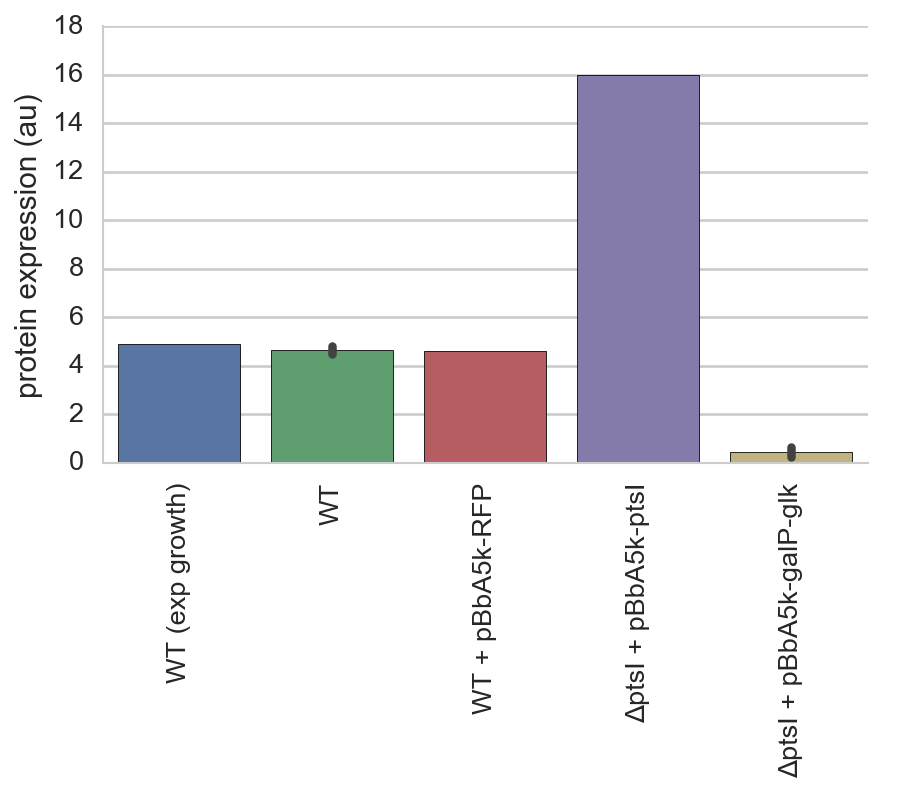


Supplementary Figure 4. Expression of PtsI measured by mass spectrometry in strains with engineered glucose uptake systems. Bars are averages of at least two biological replicates. Proteomic samples were prepared as described previously (Batth et al., 2012) and analyzed as described by (González Fernández-Niño et al., 2015).

**References**

Batth, T.S., Keasling, J.D., and Petzold, C.J. (2012). Targeted Proteomics for Metabolic Pathway Optimization. In Fungal Secondary Metabolism, N.P. Keller, and G. Turner, eds. (Totowa, NJ: Humana Press), pp. 237–249.

González Fernández-Niño, S.M., Smith-Moritz, A.M., Chan, L.J.G., Adams, P.D., Heazlewood, J.L., and Petzold, C.J. (2015). Standard flow liquid chromatography for shotgun proteomics in bioenergy research. Front. Bioeng. Biotechnol. *3*, 44.
